# Detection of CMY-type beta-lactamases in *Escherichia coli* isolates from paediatric patients in a tertiary care hospital in Mexico

**DOI:** 10.1186/s13756-020-00840-4

**Published:** 2020-10-29

**Authors:** Jocelin Merida-Vieyra, Agustín De Colsa-Ranero, Yair Calderón-Castañeda, Alejandra Aquino-Andrade

**Affiliations:** 1 Molecular Microbiology Laboratory, Instituto Nacional de Pediatria, Insurgentes Sur 3700C, Insurgentes Cuicuilco, Coyoacan, 04530 Mexico City, Mexico; 2 Department of Paediatric Infectious Diseases, Instituto Nacional de Pediatria, Mexico City, Mexico; 3 Clinical Bacteriology Laboratory, Instituto Nacional de Pediatria, Mexico City, Mexico

**Keywords:** CMY beta-lactamases, AmpC, *E. coli*, Paediatrics, Mexico

## Abstract

**Background:**

The aim of this study was to detect CMY-type beta-lactamases in *E. coli* isolates obtained from paediatric patients.

**Methods:**

In total, 404 infection-causing *E. coli* isolates resistant to third and fourth generation cephalosporins (3GC, 4GC) were collected from paediatric patients over a 2 years period. The identification and susceptibility profiles were determined with an automated microbiology system. Typing of *bla*_CMY_ and other beta-lactamase genes (*bla*_TEM_, *bla*_SHV_, *bla*_CTX-M_, *bla*_VIM_, *bla*_IMP_, *bla*_KPC_, *bla*_NDM_, *bla*_OXA_ and *bla*_GES_) was realized by PCR and sequencing. Phenotypic detection of AmpC-type enzymes was performed using boronic acid (20 mg/mL) and cloxacillin (20 mg/mL) as inhibitors, and the production of extended-spectrum beta-lactamases was determined with the double-disk diffusion test with cefotaxime (CTX) and ceftazidime (CAZ) discs alone and in combination with clavulanic acid. The CarbaNP test and modified carbapenem inhibition method (mCIM) were used for isolates with decreased susceptibility to carbapenems. The clonal origin of the isolates was established by pulsed-field gel electrophoresis (PFGE), phylotyping method and multilocus sequence typing.

**Results:**

CMY-type beta-lactamases were detected in 18 isolates (4.5%). The allelic variants found were CMY-2 (n = 14) and CMY-42 (n = 4). Of the *E. coli* strains with CMY, the AmpC phenotypic production test was positive in 11 isolates with cloxacillin and in 15 with boronic acid. ESBL production was detected in 13 isolates. Coexistence with other beta-lactamases was observed such as CTX-M-15 ESBL and original spectrum beta-lactamases TEM-1 and TEM-190. In one isolate, the CarbaNP test was negative, the mCIM was positive, and OXA-48 carbapenemase was detected. Phylogroup A was the most frequent (n = 9) followed by B2, E and F (n = 2, respectively), and through PFGE, no clonal relationship was observed. Eleven different sequence types (ST) were found, with ST10 high-risk clone being the most frequent (n = 4). Seventy-two percent of the isolates were from health care-associated infections; the mortality rate was 11.1%.

**Conclusions:**

This is the first report in Mexico of *E. coli* producing CMY isolated from paediatric patients, demonstrating a frequency of 4.5%. In addition, this is the first finding of *E. coli* ST10 with CMY-2 and OXA-48.

## Background

Of the family *Enterobacteriaceae*, *Escherichia coli* is one of the main causative agents of infections, both in the hospital environment and in the community [1]. In *E. coli*, the main mechanism of resistance to beta-lactam antibiotics is the production of beta-lactamases [2]. Among the different types of beta-lactamases, the AmpC-type has emerged as an important group of enzymes [3]. AmpC types are present in the chromosome of some enterobacteria but can pass to mobile genetic elements, such as plasmids, and be transferred horizontally between different species. These enzymes confer resistance to oxyimino-cephalosporins (ceftazidime, cefotaxime), cephamycins (cefoxitin) and monobactams (aztreonam). However, this spectrum of hydrolysis can be extended and affect fourth generation cephalosporins (4GC) (cefepime) [4]. Eight families of plasmid AmpC have been described based on differences in the amino acid sequence: CMY (cephamycin), FOX (cefoxitin), ACC (Ambler class C), LAT (latamoxef), MIR (Miriam hospital in Providence), ACT (AmpC type), MOX (moxalactam), and DHA (Dhahran hospital in Saudi Arabia) [3, 5]. Of these groups, CMY-2 is the most common type in *E. coli* and has been reported in Asia, Europe and North America [6–9]. In 2014, as part of the International Network for Optimal Resistance Monitoring (INFORM) program, 2,813 *E. coli* isolates were collected in 69 hospital centres in the USA, and CMY-2 was reported in 74 isolates (2.6%) [10]. In the Study for Monitoring Antimicrobial Resistance Trends (SMART) performed in 12 countries of the Asia–Pacific region in the 2008–2014 period, CMY-2 was reported in 1739 *E. coli* isolates at a frequency of 10.2% (n = 178) [11]. There have been reports of CMY-2 in Latin America: Argentina (0.9%), Colombia (3.5%) and Brazil (0.5%) [12–14]. In Mexico, only one study has reported *E. coli* producing CMY, causing a urinary tract infection in an adult patient [15]. Other studies have reported the presence of these enzymes in isolates obtained from animal origin, such as dogs (11.3%), sheep (0.64%) and turtles (9.8%) [16–18]. However, there is no information on the molecular or epidemiological characteristics of *E. coli* isolates producing CMY-type beta-lactamases in the Mexican paediatric population.

The aim of this study was to detect CMY-type beta-lactamases in isolates from *E. coli* obtained from paediatric patients attended at a tertiary care hospital.

## Methods

### Study site

The National Institute of Paediatrics (INP) is a tertiary care and teaching hospital in Mexico City with 235 beds and 40 medical subspecialties. In 2018, the INP had 6072 discharges.

### Isolates

In total, 404 non-duplicate 3GC and/or 4GC resistant *E. coli* isolates were collected from paediatric patients (0 to < 18 years) from the National Institute of Paediatrics (INP) over a 2-years period (February 2013–January 2015). These isolates were obtained from various clinical samples causing confirmed infections. We reviewed the medical files in order to collect clinical information such as: date of admission, gender, age, antibiotic treatments, among others. An infection was considered HAI if it appeared on or after the 3rd day of admission, according to the definitions of the Centers for Disease Control and Prevention (CDC) [19]. The strains were identified using the Phoenix Automated Microbiology System® (Becton Dickinson, New Jersey, USA).

### Antimicrobial susceptibility profiles of *E. coli* isolates

The susceptibility profile of the isolates was determined using the Phoenix® automated system (Becton Dickinson, New Jersey, USA). The tested antibiotics were ceftriaxone (CRO), ceftazidime (CAZ), cefepime (FEP), imipenem (IMP), meropenem (MEM) and ertapenem (ETP). The minimum inhibitory concentration (MIC) to cefoxitin was determined using the broth microdilution method following the guidelines of M07 of the Clinical and Laboratory Standards Institute [20]. The interpretation of the results was performed in accordance with CLSI document M100 [21].

### Molecular detection of ***bla***_CMY_ and other beta-lactamase genes

Total DNA extraction was performed using the QIAamp® DNA Mini Kit (QIAGEN, Hilden, Germany) following the manufacturer's instructions. Detection of *bla*_CMY_ and other beta-lactamases (*bla*_CTX-M-1_, *bla*_CTX-M-2_, *bla*_CTX-M-9_, *bla*_CTX-M8/25_, *bla*_TEM_ and *bla*_SHV_) was performed by end-point monoplex PCR. For the isolates with decreased susceptibility or resistance to carbapenems, carbapenemase genes were amplified; multiplex PCR (*bla*_VIM_, *bla*_IMP_ and *bla*_KPC_), and *bla*_NDM_, *bla*_OXA-48_ and *bla*_GES_ were individually amplified. We used previously published primers [22–25]. The reaction was performed in an AB9700 thermocycler (Applied Biosystems, Foster City, CA, USA) using AmpliTaq Gold® 360 MasterMix (Applied Biosystems, Foster City CA, USA). The fragments obtained were purified with a QIAquick PCR purification kit (QIAGEN, Hilden, Germany) and sequenced on a 3500 XL system (Applied Biosystems, Foster City, CA, USA). The sequences were analysed with the blastn program [26, 27], and multiple alignments were made with the BioEdit v7.2 program (Ibis Biosciences, Carlsbad CA, USA) to determine the beta-lactamase subtype.

### Phenotypic tests for AmpC, ESBL and carbapenemases

The phenotypic detection of AmpC enzymes was performed with double-disc synergy using ceftazidime disks (Becton Dickinson, New Jersey, USA) alone and in combination with cloxacillin (20 mg/mL) and boronic acid (20 mg/mL) (Sigma Aldrich, Milwaukee, WI). A test was considered positive if there was an increase in the inhibition diameter ≥ 5 mm of the ceftazidime in the presence of cloxacillin or boronic acid compared to the diameter of the ceftazidime without inhibitor [28].

The phenotypic detection of ESBL was performed by the combined disc method, using disks of cefotaxime (30 μg) and ceftazidime (30 μg) alone and in combination with clavulanic acid (30 μg/10 μg) (Becton Dickinson, New Jersey, USA) following the guidelines of the CLSI The presence of ESBL was confirmed by an increase of > 5 mm in the diameter of the zone of inhibition for any agent tested in combination with clavulanic acid compared with the diameter of the zone of inhibition for the agent alone [21].

Isolates with decreased susceptibility or resistance to carbapenems (IMP, MEM, ETP) were tested for carbapenemases production using CarbaNP and mCIM according to the guidelines of the CLSI [21].

### Molecular typing

The phylogenetic group of the isolates was obtained with the Clermont method [29]. The clonal relationship was determined using PFGE following the protocol for *E. coli* O157:H7 from PulseNet (CDC, Atlanta, GA) [30]. The *Salmonella enterica* serotype Braenderup ATCC BAA-664 strain was used as a molecular size marker. The band patterns were interpreted using Tenover criteria [31]. The ImageLab v5.2.1 program (Bio-Rad, Hercules, CA, USA) was used to create a 0/1 matrix, and DendroUpGMA (https://genomes.urv.cat/UPGMA/) and MEGA-X programs [32] were used to construct the dendrogram.

For detection of the *E. coli* ST131-O25b clone, *trpA*, *pabB* and *rfb* genes were amplified [33].

The MLST technique was performed by amplifying seven housekeeping genes (*adk*, *fumC*, *gyrB*, *icd*, *mdh*, *purA* and *recA)* using primers and conditions previously reported [34]. The sequences were analysed using the database available at https://enterobase.warwick.ac.uk/species/index/ecoli.

## Results

### Detection of ***bla***_CMY_ and other beta-lactamase genes

Of the 404 *E. coli* isolates, CMY-type beta-lactamases were detected in 18 (4.5%). The susceptibility profile of the isolates with CMY was FEP 22.3% (n = 4), IMP 94.4% (n = 17), MEM 94.4% (n = 17) and ETP 77.7% (n = 14); three isolates were intermediate to CAZ, one was to FOX and none was susceptible to ceftriaxone. The allelic variants found were CMY-2 (n = 14) and CMY-42 (n = 4). In 15 isolates, coexistence with other beta-lactamase genes was observed such as CTX-M-15 ESBL, TEM-1 and TEM-190 OSBL and OXA-48 carbapenemase. The genes *bla*_SHV_, *bla*_CTX-M-2_, *bla*_CTX-M-9_, *bla*_CTX-M-8/25,_ *bla*_GES_, *bla*_KPC_, *bla*_NDM_, *bla*_VIM_, *bla*_IMP_ were not detected (Table 1).

**Table 1:** Microbiological and molecular characteristics of the CMY-producing *E. coli* isolates

Key	MIC (μg/mL)							CAZ CLOX	CAZ BOR	AmpC type	ESBL test	Other enzymes	PFGE	ST131-O25b clone	Phylogroup	MLST	CC
	CRO	CAZ	FEP	FOX	IMP	MEM	ETP										
Ec 24	> 32	≥ 16	≥ 16	128	≤ 1	≤ 1	1	−	+	CMY-2	+	CTX-M-15	NT	−	D	**ST405**	405
Ec 35	> 32	≥ 16	≥ 16	> 128	≤ 1	≤ 1	≤ 0.5	−	+	CMY-2	+	CTX-M-79	NR	−	F	ST457	ND
Ec 41	> 32	≥ 16	≥ 16	64	≤ 1	≤ 1	≤ 0.5	−	−	CMY-2	+	TEM-1, CTX-M-15	NR	−	F	**ST648**	648
Ec 55	> 32	≥ 16	≥ 16	> 128	≤ 1	≤ 1	≤ 0.5	+	+	CMY-2	+	CTX-M-15	NR	−	A	**ST10**	10
Ec 65	> 32	≥ 16	≥ 16	128	≤ 1	≤ 1	≤ 0.5	+	+	CMY-2	+	CTX-M-15	NR	−	B2	ST12	12
Ec 109	> 32	≥ 16	≥ 16	16	≤ 1	≤ 1	≤ 0.5	−	−	CMY-2	+	CTX-M-15	NR	−	A	**ST10**	10
Ec 223	> 32	8	≥ 16	128	≤ 1	≤ 1	≤ 0.5	−	+	CMY-2	+	CTX-M-15	NR	+	B2	**ST131**	ND
Ec 278	> 32	≥ 16	≤ 2	128	≤ 1	≤ 1	≤ 0.5	+	+	CMY-2	−	Absent	NR	−	A	ST168	168
Ec 394	> 32	≥ 16	≥ 16	> 128	≤ 1	≤ 1	≤ 0.5	+	+	CMY-42	−	TEM-190	NR	−	A	**ST1284**	10
Ec 445	> 32	≥ 16	≥ 16	128	≥ 4	≥ 4	≥ 2	−	−	CMY-2	+	CTX-M-15, OXA-48	NR	−	A	**ST10**	10
Ec 480	> 32	≥ 16	≥ 16	> 128	≤ 1	≤ 1	≤ 0.5	+	+	CMY-42	−	Absent	NT	−	A	ST361	ND
Ec 481	> 32	≥ 16	≥ 16	128	≤ 1	≤ 1	≤ 0.5	+	+	CMY-42	+	TEM-190	NR	−	C	ST23	23
Ec 599	16	≥ 16	≤ 2	64	≤ 1	≤ 1	≤ 0.5	−	+	CMY-2	−	TEM-1	NR	−	A	ST101	101
Ec 668	> 32	≥ 16	≥ 16	128	≤ 1	≤ 1	1	+	+	CMY-42	+	CTX-M-15	NR	−	A	**ST10**	10
Ec 678	8	8	≤ 2	128	≤ 1	≤ 1	≤ 0.5	+	+	CMY-2	−	Absent	NR	−	E	ST350	350
Ec 686	> 32	≥ 16	≥ 16	128	≤ 1	≤ 1	1	+	+	CMY-2	+	CTX-M-15	NR	−	D	**ST69**	69
Ec 689	8	8	≤ 2	64	≤ 1	≤ 1	≤ 0.5	+	+	CMY-2	−	TEM-1	NR	−	A	ND	ND
Ec 714	> 32	≥ 16	≥ 16	64	≤ 1	≤ 1	≤ 0.5	+	+	CMY-2	+	CTX-M-15	NR	−	Clade I	ND	ND

In **bold** are the high-risk clone

*MIC* minimum inhibitory concentration, *CRO* ceftriaxone, *CAZ* ceftazidime, *FEP* cefepime, *FOX* cefoxitin, *IMP* imipenem, *MEM* meropenem, *ETP* ertapenem, *CLOX* cloxacillin, *BOR* boronic acid, *ESBL* extended-spectrum beta-lactamases, *PFGE* pulsed-field gel electrophoresis, *MLST* multilocus sequence typing, *CC* clonal complex, *NT* non-typeable, *ND* not determined, *NR* not related

### Phenotypic tests

The AmpC phenotypic production test was positive in 11 isolates with cloxacillin (61.1%) and 15 isolates with boronic acid (83.3%); 13 isolates were positive for the ESBL confirmatory phenotypic test. Of the four strains of *E. coli* with decreased susceptibility to ertapenem, one was negative for the CarbaNP test and positive for mCIM (Ec445). The remaining three strains were negative for both tests (Ec24, Ec668 and Ec686) (Table 1).

### Molecular typing

**Fig. 1:**
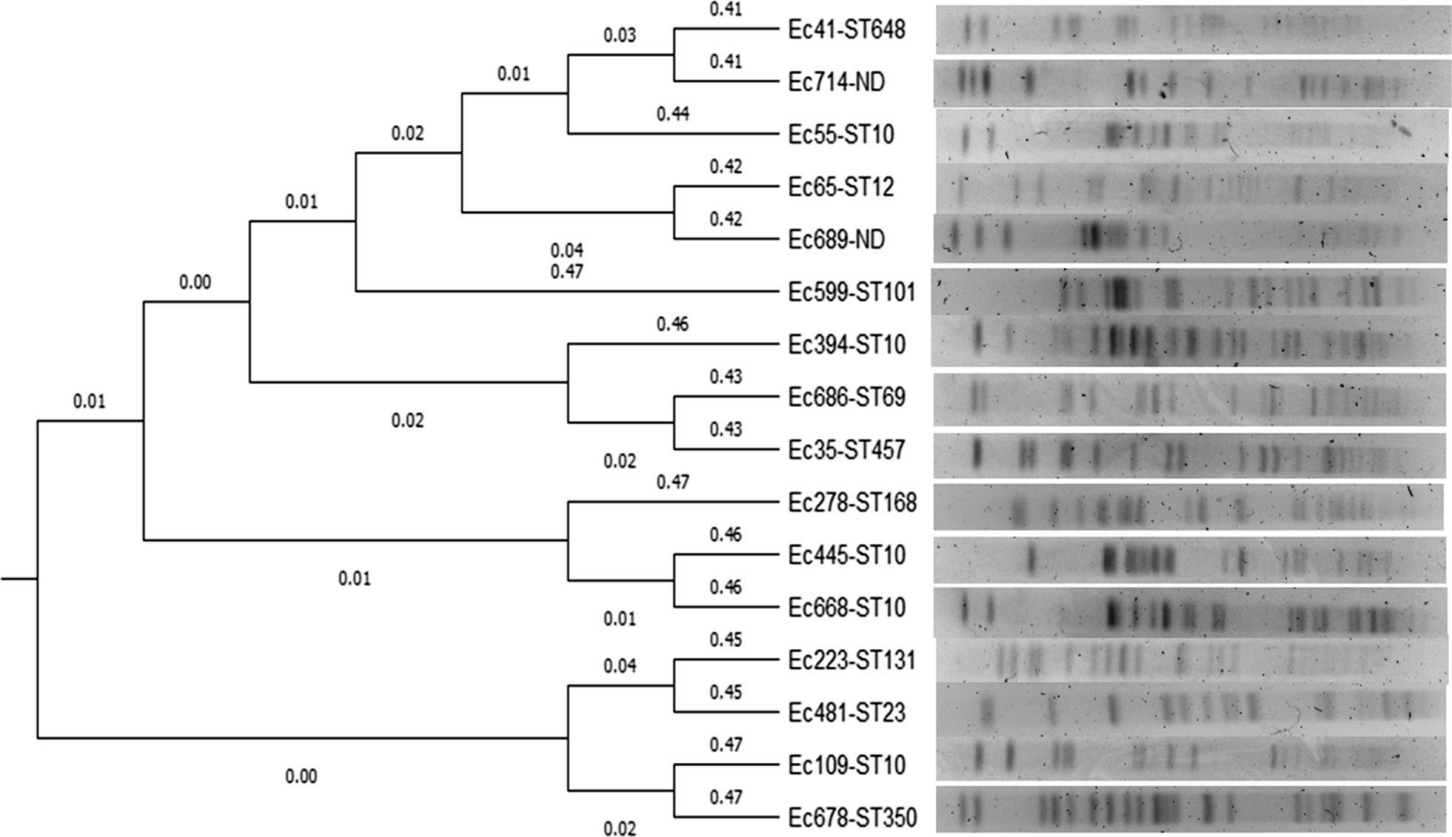
Dendrogram obtained by UPGMA (unweighted pair group method with arithmetic mean). Different pulsotypes of the CMY producing *E. coli* isolates are shown

Regarding molecular typing in the CMY-producing *E. coli* isolates, phylogroup A was the most frequent (six isolates with CMY-2 and three with CMY-42) (Table 1). Using PFGE, no clonal relationship was observed between isolates, two of which were non-typable by this technique (Fig. 1). Eleven different STs were found: ST10 (n = 5), three isolates with CMY-2 and two with CMY-42 (Table 1). One isolate corresponded to ST131-O25b clone (Ec223). The known high-risk clones ST10, ST69, ST405 and ST648 were detected. It was not possible to assign the ST in two isolates.

Regarding epidemiological analysis of patients with CMY-producing *E. coli*, 83% had comorbidities, 56% were male, and the median age was 3.5 years (range 0.6–14 years). Seventy-two percent of the isolates were from health care-associated infections. The main sample was urine (50%), followed by secretion (wound and intra-abdominal, 22.2%) and blood (16.7%). Eleven patients received 3GC as empirical treatment. Ten patients received carbapenems as definitive treatment, and just two received cefepime. The largest number of isolates was reported in 2014 (n = 10). Two patients died from septic shock (Table 2).

**Table 2:** Clinical characteristics of patients with CMY-producing *E. coli* isolates

Key	Gender	Age	Date	Comorbidity	Infection	Sample	Empirical treatment	Definitive treatment	Acquisition	Outcome
Ec 24	F	Preschooler	Feb-2013	Idiopathic ascites	UTI	Urine	FEP	FEP	H	Alive
Ec 35	F	Preschooler	Mar-2013	Aplastic anaemia	UTI	Urine	CRO	MEM	H	Alive
Ec 41	M	Adolescent	Apr-2013	Common variable immunodeficiency	Pneumonia	Bronchial aspirate	CRO	MEM/PTZ	H	Dead Septic shock
Ec 55	M	Infant	Apr -2013	Primary immunodeficiency	Perianal abscess	Secretion	CRO	CRO	H	Alive
Ec 65	F	Infant	May-2013	Down syndrome	UTI	Urine	MEM	MEM	H	Alive
Ec 109	F	School-age	Jun-2013	Neurogenic bladder	UTI	Urine	CFM	CFM	C	Alive
Ec 223	F	Infant	Oct-2013	Down syndrome Congenital heart disease	UTI	Urine	CRO	ETP	H	Alive
Ec 278	F	Preschooler	Jan-2014	Pyelonephritis	Bacteraemia	Blood	CRO	MEM	H	Alive
Ec 394	M	School-age	Apr-2014	Appendectomy	Wound infection	Intra-abdominal and wound secretion	CRO	CFM	C	Alive
Ec 445	M	Adolescent	Aug-2014	ALL	Perianal abscess	Secretion	MEM, PTZ	MEM/AK	H	Alive
Ec 480	F	Infant	Aug-2014	Neurogenic bladder	UTI	Urine	CRO	ETP	C	Alive
Ec 481	M	Adolescent	Sep-2014	Aplastic anaemia	Septic shock	Blood	MEM	MEM	H	Dead Septic shock
Ec 599	F	Adolescent	Nov-2014	None	UTI	Urine	CRO	CRO	H	Alive
Ec 668	M	School-age	Dec-2014	Uretero-pyelocaliceal stenosis	UTI	Urine	NIF	NIF	C	Alive
Ec 678	M	Infant	Dec -2014	Anorectal malformation	UTI	Urine	CRO	CRO	C	Alive
Ec 686	M	Infant	Dec -2014	Congenital hydrocephalus	Ventriculitis	CSF	MEM	MEM	H	Alive
Ec 689	M	Adolescent	Dec-2014	Right femur osteosarcoma	Catheter-associated infection	Blood	CRO	FEP	H	Alive
Ec 714	M	School-age	Jan-2015	Hydrocephalus	Cerebral abscess	Secretion	MEM	MEM	H	Alive

*ALL* acute lymphoblastic leukaemia, *UTI* urinary tract infection, *CSF* cerebrospinal fluid, *FEP* cefepime, *CRO* ceftriaxone, *MEM* meropenem, *CFM* cefixime, *PTZ* piperacillin/tazobactam, *NIF* nitrofurantoin, *ETP* ertapenem, *AK* amikacin, *H* hospital, *C* community

## Discussion

This study represents the first report in Mexico of *E. coli* isolates producing CMY in the paediatric population. Previous studies in our country have documented the presence of these enzymes only in adult patients and animals [16–18]. In this study, the frequency of *bla*_CMY_ in *E. coli* was 4.5%, like that reported in three paediatric hospitals in Chicago, USA. (4.4%) [35]. Another study in the same country found 27.9% of *E. coli* isolates with CMY [36]. In contrast, in Chinese children, CMY was reported only in 1% of isolates [37]. Although ESBL, specifically CTX-M-15, are predominant in *E. coli*, in recent years, there have been an increase in reports of strains producing AmpC, mainly CMY.

CMY-2 is the most frequent AmpC-type beta-lactamase in *E. coli*, according to reports from countries, such as Spain and Japan [38, 39]. In our study, this subtype was present in 77.8% of isolates with CMY, and the rest was CMY-42. This variant has been described mainly in India and Egypt [40, 41]. The selection pressure has caused the emergence of variants with hydrolytic activity towards extended-spectrum cephalosporins.

Resistance to cefoxitin, together with sensitivity to cefepime, has been used as a marker of AmpC-type enzyme production. However, in this study, an isolate with CMY presented intermediate sensitivity to cefoxitin, and 14 (78%) were resistant to cefepime. Twelve isolates (67%) were positive for the ESBL phenotypic test, higher than reported in the literature (9.9–36%) [42], which explains the resistance to cefepime. In two isolates with a negative ESBL test (Ec394, Ec480), CMY-42 could have been responsible for resistance to cefepime. The detection of these enzymes is complicated due to the simultaneous production of ESBL and AmpC in an isolate, which makes difficult the interpretation of the antimicrobial susceptibility test.

Due to the lack of standardized methods for the phenotypic detection of these enzymes, their prevalence may be underestimated. One of the phenotypic methods for this purpose is based on the use of cloxacillin and boronic acid as inhibitors with a specificity of 50–90% [43, 44]. In this study, boronic acid was a better inhibitor (83.3%) than cloxacillin (61.1%). In contrast, other reports have indicated greater sensitivity using cloxacillin (77–94%) [28, 41]. However, three isolates with CMY were negative for the phenotypic test with both inhibitors. Given the limitations of phenotypic tests and the lack of standardized methods by international guidelines, such as CLSI or EUCAST, molecular methods, such as PCR, should be considered for the detection of these enzymes.

The presence of ESBL and CMY was observed in 11 isolates (61.1%), all with CTX-M group enzymes, mainly CMY-2 with CTX-M-15 (n = 9). In Japan, CTX-M-15 was detected with CMY in 72% of *E. coli* strains [45]. In one isolate (Ec394) with a positive ESBL test, only TEM-190 was detected, which is not an ESBL, thus the ESBL profile may have been due to another enzyme that was not determined in this work. Although the frequency of CMY is lower than that of ESBL, it is clinically significant because when combined with the loss of porins (OmpC, OmpF), it can confer resistance to carbapenems, one of the last-line antibiotics for the treatment of infections [46]. On the other hand, CMY enzymes have relevance in resistance to recently introduced antimicrobials, for instance in a study carried out in centers in USA; two *E. coli* isolates were found, in which genes encoding the CTX-M-15 and CMY-2-like beta-lactamases coexisted; both isolates were resistant to ceftolozane-tazobactam with MIC 64 µg/mL and 128 µg/mL, respectively [47]. The existence of these two mechanisms of resistance in the same isolate can complicate the interpretation of the antibiogram and, consequently, the proper selection of antimicrobial treatment.

In one isolate (Ec445), co-production of CMY-2 with the carbapenemase OXA-48 was detected, which represents the first report of this coexistence in Mexico. This combination was reported in the USA, in isolates obtained from dogs and cats [48]. In other studies, the simultaneous production of CMY with other carbapenemases has been reported. CMY-42 has been described in coexistence with NMD-5 in Italy [49] and Spain [50], and the presence of CMY-42 with NDM-1 and CTX-M-15 has been reported in China [51] and CMY-42 with OXA-181 in India [52]. The simultaneous production of different beta-lactamases decreases the therapeutic options for the treatment of infections.

In Malaysia and the USA, patients colonized by CMY-2-producing *E. coli* have been described at frequencies of 6.4% and 24%, respectively [53, 54]. In the Netherlands, it was reported that 1.1% of adult carriers were colonized by *E. coli* with CMY-2 [55]. In Mozambique, *E. coli* was detected with CMY-2 in 20% of university students [56]. The presence of these enzymes has also been documented in dogs and cats, with frequencies ranging from 3.8 to 85% [57, 58]. In addition, the coexistence of CMY-2 with the *mcr*-1 gene has been detected in poultry, in Germany [59] and in food-producing animals, in Portugal [60]. These studies indicate that people and animals are reservoirs for the dissemination of *E. coli* strains with this type of enzyme or can transfer resistance genes to other species.

Fifty percent of *E. coli* isolates with CMY were classified into commensal phylogroup A (33.3% CMY-2, 16.7% CMY-42), in contrast to that reported in Spain, where pathogenic phylogroup B2 was the most frequent among *E. coli* with CMY-2 [61]. Other studies in New Zealand and the USA, have reported D as the main phylogroup [62, 63]. In France, a higher frequency was reported in commensal phylogroups (A, B1 and C) than in pathogenic phylogroups (B2, D and F) [64]. Thus, CMY presents a heterogeneous distribution among phylogroups, and commensal *E. coli* can acquire resistance genes as well as the capacity to cause infections.

There are reports indicating that the dissemination of *E. coli* isolates producing CMY is non-clonal [61, 62]. In this study, 11 different STs were obtained, and the main was ST10, which is considered a high-risk clone. This ST has been reported in isolates from humans and in chicken meat in Germany [59]. High-risk clones ST69, ST405 and ST648 were also detected. ST69 with CMY-2 has been described in dairy cows in the Czech Republic [65] and in an *E. coli* strain with *mcr*-1 obtained from a urinary tract infection case, in England [66]. ST405 and ST648 with CMY-2 have been described in strains isolated from pets in the USA coexisting with OXA-48 [48]. *E. coli* ST457 with CMY-2 has been reported in molluscs and poultry in Brazil [67, 68] and in an isolate obtained from a paediatric patient in the USA [69]. *E. coli* ST23 with CMY-2 has been detected in chicken faeces and meat in Denmark [70], and a strain of *E. coli* ST361 with CMY-42 from the urinary tract was reported in India [40]. In this study, these two clones (Ec480-ST361 and Ec481-ST23) had CMY-42. On the other hand, *E. coli* ST12 with CMY-2 was reported in poultry in Italy [71], *E. coli* ST350 in chicken carcasses in Brazil [72], *E. coli* ST101 with CMY-42 was described in a paediatric patient with bacteraemia [69] and *E. coli* ST1284 with CMY-2 in a dog with skin and soft tissue infection [73]. Due to the diversity of ST found in this study and those reported in other studies, the dissemination of CMY enzymes seems not to be related to a specific lineage of *E. coli*, but rather to the transfer of mobile genetic elements. Likewise, there are several reservoirs for these beta-lactamases, such as animals, food and aquatic environments.

In this study, only one isolate with CMY-2 belonged to the *E. coli* ST131-O25b clone, which is responsible for the global dissemination of CTX-M-15. This ST with CMY has been reported in strains that cause urinary tract infections and bacteraemia [62, 74, 75] and in strains obtained from chickens in Germany [59]. Although there are few reports of *E. coli* ST131-O25b with CMY, it is important to surveillance the distribution of this clone due to its epidemic potential.

Different risk factors associated with the acquisition of *E. coli* with AmpC, such as comorbidities, previous use of antimicrobials, use of medical devices, urinary tract abnormalities and prolonged hospital stay have been described in adult patients [76, 77] In the current study, most of the patients had comorbidities and had previously received broad-spectrum antimicrobials (3GC, 4GC and carbapenems). Three patients had urinary tract pathologies, which could have caused greater susceptibility to urinary tract infections. Two patients died from septic shock derived from CMY-producing *E. coli* infections.

## Conclusions

This study represents the first report of *E. coli* producing CMY in the paediatric population in Mexico, with a frequency of 4.5%. The dissemination of these enzymes in our study was associated with high clonal diversity. A high proportion of *E. coli* producing CMY also harbour ESBL enzymes. The difficulty of detecting CMY only by phenotypic tests, makes the use of molecular techniques essential.

Due to the emergence of high-risk *E. coli* clones in our hospital, it is necessary to maintain an epidemiological surveillance programme that allows the timely detection of these strains to prevent outbreaks.

In addition, this is the first report of *E. coli* ST10 with CMY-2 and OXA-48 in Mexico.

## Data Availability

All the data supporting our findings is contained within the manuscript.

## Abbreviations

ESBL: Extended-spectrum beta-lactamases
OSBL: Original spectrum beta-lactamases;
mCIM: Modified carbapenem inhibition method
PFGE: Pulsed-field gel electrophoresis
MLST: Multilocus sequence typing
3GC: Third generation cephalosporins
4GC: Fourth generation cephalosporins
CMY: Cephamycin
FOX: Cefoxitin
ACC: Ambler class C
LAT: Latamoxef
MIR: Miriam hospital in Providence
ACT: AmpC type
MOX: Moxalactam
DHA: Dhahran hospital in Saudi Arabia
MIC: Minimum inhibitory concentration
UPGMA: Unweighted pair group method with arithmetic mean
